# An Explainable Evolving Fuzzy Neural Network to Predict the k Barriers for Intrusion Detection Using a Wireless Sensor Network

**DOI:** 10.3390/s22145446

**Published:** 2022-07-21

**Authors:** Paulo Vitor de Campos Souza, Edwin Lughofer, Huoston Rodrigues Batista

**Affiliations:** 1 Institute for Mathematical Methods in Medicine and Data Based Modeling, Johannes Kepler University Linz, 4040 Linz, Austria; edwin.lughofer@jku.at; 2 School of Informatics, Communication and Media, University of Applied Sciences Upper Austria Hagenberg, 4232 Hagenberg im Mühlkreis, Austria; huoston.rodrigues@fh-hagenberg.at

**Keywords:** evolving fuzzy neural networks, interpretability, k barriers, intrusion detection, wireless sensor networks

## Abstract

Evolving fuzzy neural networks have the adaptive capacity to solve complex problems by interpreting them. This is due to the fact that this type of approach provides valuable insights that facilitate understanding the behavior of the problem being analyzed, because they can extract knowledge from a set of investigated data. Thus, this work proposes applying an evolving fuzzy neural network capable of solving data stream regression problems with considerable interpretability. The dataset is based on a necessary prediction of k barriers with wireless sensors to identify unauthorized persons entering a protected territory. Our method was empirically compared with state-of-the-art evolving methods, showing significantly lower RMSE values for separate test data sets and also lower accumulated mean absolute errors (MAEs) when evaluating the methods in a stream-based interleaved-predict-and-then-update procedure. In addition, the model could offer relevant information in terms of interpretable fuzzy rules, allowing an explainable evaluation of the regression problems contained in the data streams.

## 1. Introduction

Evolving fuzzy neural networks are hybrid models that combine artificial neural networks with fuzzy logic [1,2] while being able to incrementally adapt their parameters and evolve their internal structure with newly recorded data (typically in the form of stream samples) [3]. Due to their universal approximation capabilities [2], inherited from classical fuzzy systems [4], they are able to solve complex data-stream mining problems with high precision while, at the same time, being able to extract knowledge from data and offer interpretable insights into this knowledge in form of (fuzzy) rules [5].

A current problem that has been the subject of research in the scientific community is the use of artificial intelligence and border protection in countries [6]. Numerous research approaches incorporate predictions of elements needed to secure a country’s borders, primarily linked to wireless sensors for detecting people crossing unduly from one country to another [7]. Countries with large territorial extensions can use these resources to facilitate the control of incoming people. Therefore, the use of intelligent techniques to rationalize the process and make it more effective is well-justified.

However, many models used in this context offer no other way to interpret the results, especially when evaluating the flow data or examining the behavior of the model(s) over time [3]. To fill this gap, methods with the ability to extract knowledge from data streams in an incremental, evolving manner have been proposed in the literature, while offering a joint interpretability of the functioning of the extracted models in the evaluation of new data, basically for stream classification problems in [8,9], or see also [10,11].

This paper aims to present the extension of an evolving fuzzy neural network (ENFS-Uni0) [8] to act on stream regression problems (named here as ENFS-Uni0-reg).In this work, we intend to explain how the interpretability of the results with the interpretability of the model’s behavior can help the analysis of knowledge contained in a data stream. ENFS-Uni0-reg is an evolving model with three layers, the first being the data-fuzzification process. This method is performed by a fuzzification technique based on the scalable data density responsible for building Gaussian neurons to represent the data of a problem. The second layer includes fuzzy-logic neurons that aggregate the neurons formed in the first layer with their respective weights. Finally, the third layer of the model is represented by an artificial aggregation neural network, which is responsible for the defuzzification process. Interpretability aspects of Gaussian neuron development, assessment of problem features, and the training of interpretable consequences of rules are embedded in our approach to support an advanced interpretation of results. The interpretability of fuzzy-systems models may meet different criteria from conventional models of artificial neural networks. Therefore, practical examples of problem solving must be presented in an interpretable way according to the interpretability criteria proposed in [12] for this category of models. These examples are provided by analyzing the model’s behaviors when performing the regression tasks. Therefore, it may be possible to measure the degree of interpretability achieved by ENFS-Uni0-reg. Thereby, the main focus will be placed on a particular application scenario, which is the identification of the number of barriers in wireless connections (as is explained in more detail in the subsequent section). This paper proposes to work with the resolution of a regression problem to identify the number of barriers in wireless connections.

The first contribution of this paper is extending an interpretable evolving fuzzy-neural-network model to solve regression problems. Thereby, the main highlight of this paper is to present the principal features of interpretability that the evolving fuzzy neural networks could achieve for k-barriers identification in wireless systems. An intrinsic aspect, thereby, is that the relevancy of the final achieved readable rules could be confirmed by an expert (the data owner) of the application. Thus, a reader may be able to follow the characteristics of the interpretability, facilitating the understanding of the use of these models. Another relevant contribution is the modification of the feature weights concept proposed in our previous work for stream classification [8] in stream regression problems. This factor allows the features of regression problems to also be interpreted and incorporated into the training process of the system model.

The paper presents a theoretical reference section (Section 3) associated with the concept of k barriers for intrusion detection, evolving fuzzy models, and their respective interpretability capabilities. Section 4 offers the reader the model’s layers and training procedures. Experiments and their discussions are emphasized in Section 5 and Section 6, respectively. Finally, in Section 7, conclusions regarding the major result findings in our empirical studies are presented.

## 2. k Barriers for Intrusion Detection in Wireless Sensor Networks—State-of-the-Art Approaches

**Figure 1:**
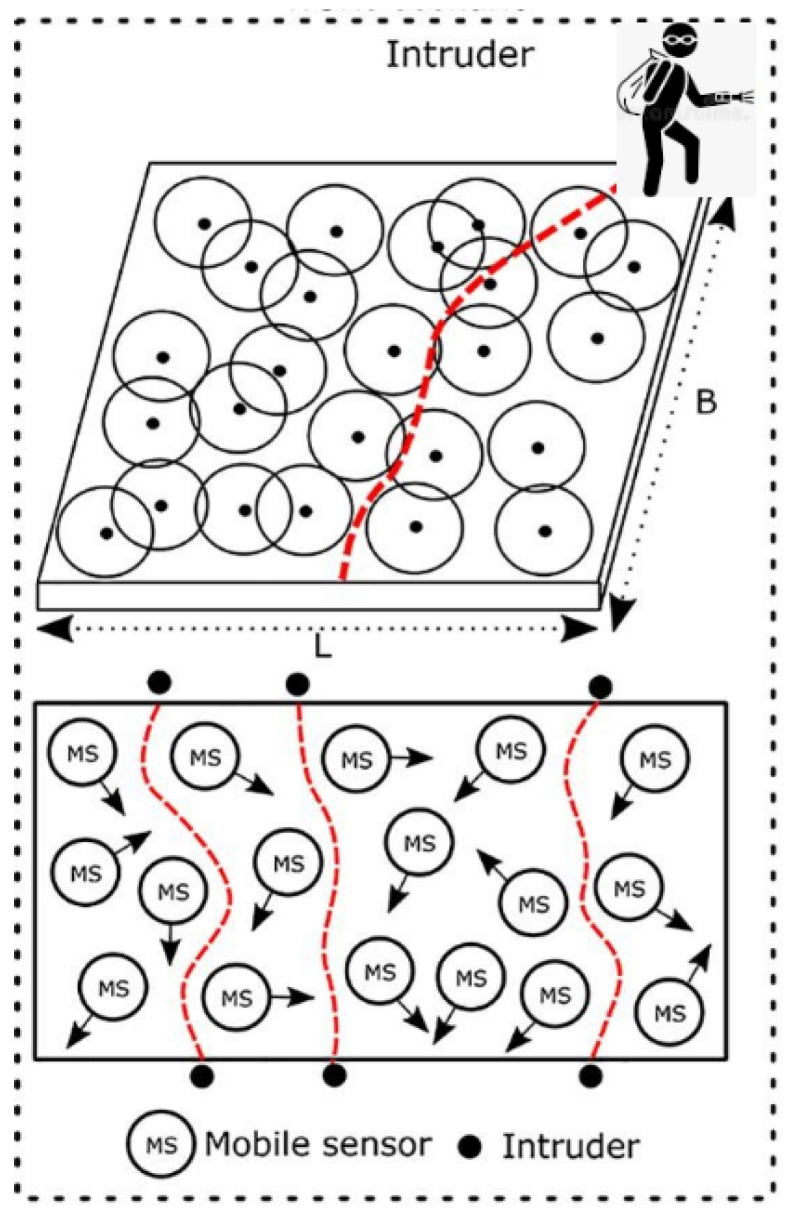
Example of sensors for identifying intruders.

Issues of territorial protection emerge as new conflicts and problems arise in modern society. Border protection interests all nations, especially those involved in armed conflicts or close to areas with a high flow of illegal products (drugs, weapons smuggling, and illegal immigrants, among others) [13]. To help control this identification of possible invaders, several sensors are used to identify suspicious movements at the borders of countries [14] (Figure 1). This technology is relatively inexpensive and can help monitor existing activities in these locations. These sensors use machine-learning concepts to facilitate the identification of adverse situations [14]. This type of technology also helps to reduce the number of human resources guarding countries with large borders. Sensors installed at borders are decentralized equipment that transmit data over a wireless network. Thus, these sensors detect possible intruders who are not allowed to enter a certain target territory [15]. Monitoring centers can act dynamically to identify them, acting quickly to avoid further problems caused by the unwanted invasion [16]. The use of machine learning to improve these identifications has been extensively discussed in the literature, thus making it a subject of great relevance and scope in science [17]. Authors have already proposed solutions to the problem using Gaussian processes [18], coverage probability in a mobile sensor network [19], low-power sensors inside the region of interest [20] and on-demand distance vector routing [21]. However, until the present moment, the interpretability of these sensors during identification has not been specified. This type of problem fits into the evaluation of streaming data, as it never knows when possible invaders will cross a country’s border.

## 3. Evolving Fuzzy Systems and Interpretability

### 3.1. Evolving Fuzzy Systems

Evolving fuzzy systems (EFS) have a significant difference compared to traditional neural network models, which is the ability to combine the advantages of fuzzy inference systems with training artificial neural network devices that adjust parameters based on the dynamics of the data [22].

The fundamental concepts of evolving fuzzy systems are their ability to process samples or blocks of data step-by-step for model building, omitting time-consuming retraining (incrementality), performing recursive parameter adjustments (customization), and adding structural elements (evolving) if the system handles newly loaded samples or has new states. EFS is also an incrementally evolving single-pass fuzzy system with online data streams/registrations with similarities between specific architectures with certain types of neural networks. They are, thus, a form of gray-box models as they combine data-driven learning with rule-based structures [22].

The key requirements for real-world applications are quick online model identification from scratch, updating and extending existing models, avoiding extrapolation, providing process reliability and robustness, and working with adjustments of the knowledge-based model extracted from the data, see [3,5] for recent surveys about successful applications of EFS.

Evolving fuzzy neural networks (EFNNs) are an extension of plain EFS by incorporating a layered structure in the form of hidden neurons in their model architecture. The hidden layers of these models vary according to the number of features in the fuzzy inference systems and the aggregations performed by the neural networks [1]. This ability of the fuzzy inference system that makes up the evolving fuzzy neural network allows a more sophisticated representation of rules (mixed AND-OR connections with smooth transitions inbetween their antecedents) through the usage of extended aggregation operators such as the uni-norms being applied to the construction of the neurons [23], which can be further associated with fuzzy rules [8]. Due to this direct association (see also Section 4), it is then possible to analyze the knowledge contained in the stream and its evolution over (timely) stream examples. Examples of evolving fuzzy neural network architectures can be seen in [9,24,25,26,27,28].

### 3.2. On-Line Criteria of Interpretability

A general definition of interpretability in machine-learning models is the degree to which a human can understand the cause of a decision or the degree to which a human can consistently predict the outcome of the decision model. [29]. Interpretable machine learning is an all-encompassing term that captures the extraction of relevant knowledge from a machine-learning model about relationships in data or learned from the model. In the context of evolving learning, some characteristics need to be modified so that the evaluation of these patterns occurs in a way that humans can understand, mainly due to their adaptive behavior [29].

The author of [12] discussed the criteria of transparency, readability, and interpretability of EFS models and their contained fuzzy rule bases, which can be largely applied also to EFNN models. To this end, some essential criteria have been defined for the evaluation of interpretability in these models, listed below [12]:

- **Distinguishability and simplicity:** Simplicity requires models with a trade off between low complexity and high accuracy in order to avoid over-fitting effects [30]. In contrast, distinguishability requires the use of structural components (rules, fuzzy sets) in a separable way (avoiding significant overlaps and redundancies [31]).
- **Consistency:** Consistency in a rule base is given when there are no conflicting, contradictory rules [32], e.g., when no rules overlap in their antecedents and consequents or when there is a significant overlap between two or more rules in their antecedents, then their consequents are also similar (overlapping). A fuzzy rule is consistent with another one if the similarity of their antecedents is lower than the similarity of their consequents [12].
- **Feature importance levels:** This capability evaluates the importance of features in the final output of the model. It allows evaluation of their impact to explain the knowledge contained in the data (input–output relations) and also may serve for the possibility to reduce the rules’ length, which, in turn, increases their transparency and understandability (see the Results section for a concrete example).
- **Rule importance levels:** estimations of the significance levels of the rules are defined by numerical values (weight or consequents of the rules) to assess the relevance of the rule for the analyzed context and, thus, its importance to the prediction capabilities of the model.
- **Interpretation of consequents:** The consequents should not be too complex (e.g., higher order polynomials or wavelet functions), but represent the essential rule output statement for the local region it represents—for instance, in classification problems, a class confidence vector indicating the confidences in the different classes for the respective local regions leads (in combination with the rules’ antecedent parts) to a direct interpretation where classes are located in the feature space and overlap. In our case of regression problems, we deal with singleton numerical output values whose direct interpretation is the number of barriers in the associated region of the rule models. Another interpretation facet is how the consequents may change during stream learning and, thus, how rules may change their ’opinion’ in their output (we provide an example in the Results section).
- **Knowledge expansion:** Evaluation of results beyond accuracy. The criteria for incorporating new knowledge, assessments, and operations with the rules fit as parameters for this measure. The rules evolution criteria also make it possible to identify an evaluation of the knowledge obtained from the model.

To be considered interpretable, an EFNN model should, ideally, meet both the criteria of high accuracy (to ensure the efficiency of the results) and the criteria discussed above. This is because a model with low predictive accuracy does not reflect well the behavior of the system. The model architecture, training, and interpretability improvement techniques are presented in the next section.

## 4. Evolving Fuzzy Neural Network for Regression Problems—ENFS-Uni0-reg

This section presents the architecture and training characteristics of the ENFS-Uni0-reg [8]. Aspects of its operation contribute to the interpretability of online problems and enable the evaluation behavior of the model when it analyzes data sets. The architecture of ENFS-Uni0-reg consists of three layers. The first two are a fuzzy inference system (responsible for extracting knowledge from the data set via IF-THEN rules). A neural-network-based layer represents the last layer, which is able to aggregate all the consequents of the fuzzy rules and convert them into the expected output (the defuzzification process) [8].

The model parameters are defined during a fuzzification process which specifies the number of fuzzy rules the system can extract from the data. This process leads to the trained Gaussian neurons with the centers and standard deviation of the clusters found using a stand-alone data-clustering technique [33] called autonomous data partitioning (ADPA). These clusters are defined by concepts of data density and empirical data operators [34]. The technique’s significant benefit is to use local mode to partition the input space in structures called data clouds, using approaches similar to the Voronoi tessellation technique. Another advantage is the objective representation of the actual distribution of data, creating clusters in arbitrary shapes. Therefore, these Gaussian neurons generated by the fuzzification process are liable for the formatting of the antecedent terms of the fuzzy rules [8]. It is worth mentioning that ENFS-Uni0 uses an extended supervised version of ADPA. In this paper, as it deals with the resolution of a regression problem, we obtained the original version of the technique proposed in [33].

This method has some steps to be performed to obtain the clusters (clouds):

- Stage 1: Ranking of the samples regarding the distance to the global mode.
- Stage 2: Detecting local maxima (local modes).
- Stage 3: Forming data clouds.
- Stage 4: Filtering local modes.

All these stages are performed in the offline phase of the model. The model proposed in this paper uses these steps to define the model’s initial architecture, since ADPA is able to autonomously extract an adequate number of rules for the correct problem (subject to density criteria) and thus defines the number of neurons (rules) for the network (omitting this as a predefined parameter). During the evolving phase, the algorithm works with the following steps:

- Stage 5: Selection of the first data sample within the data stream as the first local mode.
- Stage 6: System structure and meta-parameters update.
- Stage 7: Forming data clouds from the updated structure and parameters.
- Stage 8: Handling the outliers.

Thus, the fuzzification technique allows the evolution of neurons as new information or behaviors are noticed in the data. For details on the approach, see [33]. Each of these neurons has a weight determined by an online technique for determining the class separability criterion of the problem (feature weight separability criteria for regression problems (FWSCR)—to be explained in detail later). This approach brings advantages of interpretability to the dimensions of the problem (identifying those with greater relevance to find the values to be predicted to the model) and permitting a reduction in the rule lengths, generating compact knowledge about the problem in question [8]. These neurons and their weights are grouped in the second layer of the fuzzy logic neuron model. This neuron uses a fuzzy aggregator to combine weights and the Gaussian neuron and turn it into a single value [8]. For this, the EFNS-Uni0-reg uses the stochastic approach defined in [35], where the fuzzy neurons in the second layer are defined according to the best model architecture for solving the problem.

This skill facilitates the dynamic resolution of complex problems, allowing for knowledge extraction with flexibility [8]. The rule consequents are determined differently according to the stage in which the model executes its activities to complete the formation of fuzzy rules. The extreme-learning-machine concept [36] (using the pseudo-inverse of the Moore–Penrose matrix [37]) is applied in the offline stage. In the evolving phase of the model, the rule consequents are updated by a recursive weighted least squares technique [38] for the regression problems [8], embedding the activation levels of the rules in the current sample as weights. Rules covering a current sample better and, thus, having higher activation levels, are more intensively updated as better representing the current state of the system. The model output is obtained by a neural aggregation network with only one neuron (singleton concept in order to achieve high interpretability). This neuron, which has a linear activation function, is responsible for the model result [8]. The proposed approach has the capabilities to extract knowledge and interpret the results. The fuzzy rules formed by EFNS-Uni0-reg can be described by [8]:

(1) $$\begin{array}{c} Rule_{1}:\;If\;x_{1}\;is\;A_{1}^{1}\;with\;impact\;w_{11}\ldots \\ and/or\;x_{2}\;is\;A_{1}^{2}\;with\;impact\;w_{21}\ldots \\ Then\;y_{1}\;is\;v_{11}\\ ....Rule_{L}:\;If\;x_{1}\;is\;A_{L}^{1}\;with\;impact\;w_{1L}\ldots \\ and/or\;x_{2}\;is\;A_{L}^{2}\;with\;impact\;w_{2L}\ldots \\ Then\;y_{L}\;is\;v_{L} \end{array}$$

where **A** are the Gaussian neurons (where the membership functions of fuzzy sets, formed by the input data density through an evolving clustering method, are the activation functions of the corresponding neurons, i.e., $a_{jl}=\mu_{l}^{A}$ for *j* = 1... *N* and *l* = 1 … *L*, where *N* is the number of inputs and *L* is the number of fuzzy sets for each input) and **w** is its respective weight ($w_{il}$ (for *i* = 1... *N* and *l* = 1... *L*). The consequent values ${\vec{v}}_{.}$ are calculated in two different ways. The offline phase is based on Equation (2) and the evolving step uses Equation (5), related below.

(2) $${\vec{v}}_{k}=Z^{+}\vec{y}\;$$

(3) $$\eta ={\vec{z}}^{t}Q^{t-1}{\left(\psi +{\left({\vec{z}}^{t}\right)}^{T}Q^{t-1}{\vec{z}}^{t}\right)}^{-1}$$

(4) $$Q^{t}=\left(I_{L^{t}}-\eta^{T}{\vec{z}}^{t}\right)\psi^{-1}Q^{t-1}$$

(5) $${\vec{v}}_{k}^{t}={\vec{v}}_{k}^{t-1}+\eta^{T}\left(y_{k}^{t}-{\vec{z}}^{t}{\vec{v}}_{k}^{t-1}\right)$$

$Z^{+}=Z^{T}Z$ is the pseudo-inverse of the Moore–Penrose matrix [37] of *Z* (logic fuzzy neuron) and *y* denotes the column output. η is the current Kalman gain (row) vector, **$I_{L_{s}^{t}}$** is an identity matrix based on the number of neurons in the second layer, $L_{s}^{t}\times L_{s}^{t}$; ψ∈]0,1] denotes a possible forgetting factor, but is to 1 per default (no forgetting). *Q* denotes the inverse Hessian matrix $Q={\left(Z^{T}Z\right)}^{-1}$ and is set initially as $\omega I_{L_{s}^{t}}$, where ω = 1000. *Z* is a logic neuron vector, and this fuzzy neuron can be represented by:

(6) $$\begin{array}{c} z=\zeta_{\eta }\left(w;a\right)=\gamma_{i=1}^{n}p\left(w_{i},a_{i}\right)\\ where\;\eta =1\;or\;2\;or\;3\;or\;4\;or\;5 \end{array}$$

where ζ can represent and-neurons (η = 1), or-neurons (η = 2), uni-neurons (η = 3) null-neurons (η = 4), or uninull-neurons (η = 5), and γ represents a fuzzy operator (t-norm, t-conorm, uninorm or nullnorm) which is related to the neuron used. *p* is a function to transform the values into one unique value. For and-neurons, *p* is a t-norm; for or-neurons, a t-conorm. For uni-neurons, null-neurons and uni-nullneurons, the function *p* is expressed below:

(7) $$p\left(w,a,o\right)=wa+\bar{w}o$$

where $\bar{w}$ represents the complement of *w* and *o* is a value based on the neuron operator.

The process carried out in the third layer is also seen as a defuzzification process with an aggregation neural network composed of a single neuron. The adaptations of ENFS-Uni0 so that the model can act as a regressor are presented the following definition:

(8) $$y=\left(\sum_{j=0}^{l}\left(z_{j},v_{j}\right)\right)$$

where $z_{0}$ = 1, $v_{0}$ is the bias, and $z_{j}$ and $v_{j}$, *j* = 1, ߪ, *l* are the output of each fuzzy neuron of the second layer and their corresponding weight, respectively. As the ENFS-Uni0-reg model can act as a universal approximator of functions, it can perform precise resolutions for regression problems.

### Feature Weight Separability Criteria for Regression Problems (FWSCR)

The assessment of the changes in Gaussian neurons in the first layer can measure how the rule antecedents change over time [31]. The main difference in this paper is that this approach has always been used to distinguish classes and not regression values. However, these values can be grouped into two groups: those that are lower and higher than the average of the expected outputs. The mean in this context will be a cut-off factor to determine which values are smaller than what is considered a small-valued prediction in the model output. Conversely, values above the mean are considered a high prediction of the target to be analyzed in the regression problem. Analogously, we group the expected outputs of the regression problem as if they were a binary-pattern classification problem. This process is called discretization and it has been applied several times to solve regression problems as a classification problems [39,40,41]. The average of the expected outputs is calculated in the offline phase of definition. A transform function creates an auxiliary vector, assigning the value 0 for the values smaller than the average and 1 for the superior ones. The feature weight separability criteria for regression problems (FWSCR) is represented by:

(9) $$y_{r}=\left\{\begin{array}{c} \begin{array}{c} 1,\;if\;y\;>\Omega \end{array}\\ \begin{array}{c} -1,\;if\;y\;<\Omega \end{array} \end{array}\right.where\;\Omega =\frac{1}{n_{w}}\sum_{j=1}^{n_{w}}y_{j}$$

where $y_{r}$ is the transformed vector to be used to calculate the feature separability criteria, $n_{w}$ is the number of samples designated to calculate the mean of the values and Ω is the reference value used in the discretization process. Thus, the proposition made in [42] will be able to identify the dimensions that most contribute to a prediction of inferior and superior values of the analyzed regression problem. It is worth mentioning that the mean is also updated as new samples are evaluated by the model, using the classical incremental mean formula [43]. Other interpretable factors that the model can also measure are identifying the evolution of fuzzy rules and their respective consequents. The basic idea of the technique is still to extract feature weights based on the usage of a class separability criterion, but here, the classes will be considered as the discretization outcome performed by Equation (9) in the models’ output. Therefore, for the target regression problem, one can consider the approach analogous to an evaluation with two classes (elements greater and less than the average of the outputs). The technique defined in [42] uses the Dy–Brodley separability criterion to measure the discriminatory power of a feature set for two or more classes. It can be expressed by:

(10) $$J=\delta (S_{w}^{-1}S_{b})$$

where $\delta (S_{w}^{-1}S_{b})$ describe the sum of the diagonal elements of the matrix $S_{w}^{-1}S_{b}$[42]. $S_{b}$ indicates the dispersion matrix between classes that measure the class’s dispersion averages to the total average, and $S_{w}$ denotes the within scattering matrix that calculates the samples’ dispersion in their class averages. This overview allows the model’s user to identify moments of change in the model’s efficiency or even to measure moments in which the technique acquired new knowledge [8]. Details on how the techniques work can be seen in depth at [8]. The approach employed in this paper will be leave-one-feature-out (LOFO), which means that the lower the value of $J_{i}$ becomes, the more important feature *i* is because feature *i* was discarded from the full feature set (and (10) recalculated). Thus, and aiming for a relative importance among all features (relative to the most crucial feature, which should be assigned a value of 1), the feature weights are assigned by:

(11) $$w_{j}=1-\frac{J_{j}-\min_{i=1,\ldots ,N}J_{i}}{\max_{i=1,\ldots ,N}J_{i}}$$

Thus, feature weights are assigned by:

(12) $$w_{j}=\frac{J_{j}}{\max_{i=1,\ldots ,N}J_{i}}$$

In particular, $S_{b}$ can be updated by updating the class-wise, and the overall mean through an incremental mean formula [43]. Specific recursive covariance update formulas proposed in [44] update the covariance matrices per class with rank-1 modification for more robust and faster convergence to the batch calculation.

**Figure 2:**
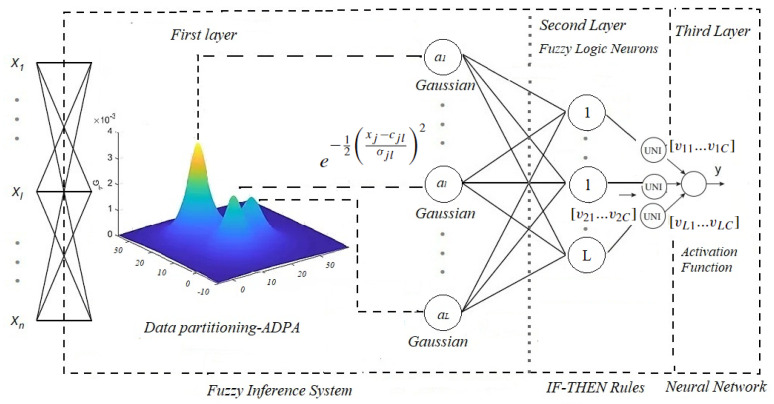
ENFS-Uni0-reg.

**Figure 3:**
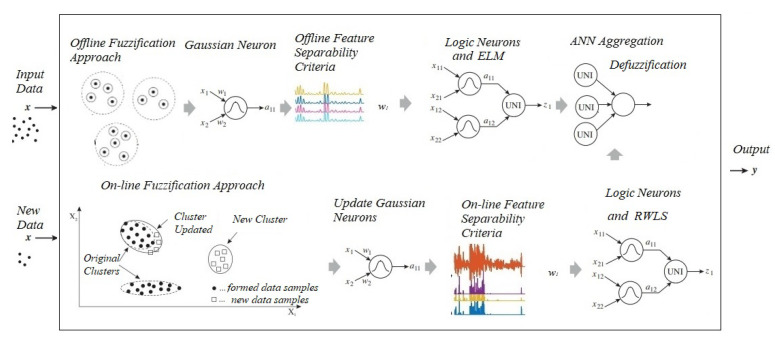
Algorithm execution steps.

The architecture of the model can be seen in Figure 2. A pseudo-code of ENFS-Uni0-reg is presented in Algorithm 1 and the organization of the steps performed in the algorithm is shown in Figure 3.

**Algorithm 1** ENFS-Uni0-reg Training and Update Algorithm

**Initial Batch Learning Phase (Input: data matrix** X **with** K **samples):**
(1) Extract *L* clouds in the first layer using the ADPA approach (*L* is automatically estimated therein).(2) Estimate center values $\vec{c}$ and widths $\vec{\sigma }$ for the *L* clouds derived from ADPA.(3) Calculate the combination (feature) weights $\vec{w}$ for neuron construction using FWSCR.(4) Construct *L* logic neurons on the second layer of the network by welding the *L* fuzzy neurons of the first layer, using uni-nullnorms concept and the centers $\vec{c}$ and widths $\vec{\sigma }$.(5)
**for** i=1,…,K **do**
   (5.1)    Calculate the regression vector $z(x_{i})$.   (5.2)    Store it as one row entry into the activation level matrix *Z*.
**end for**
(6) Extract activation level matrix *Z* according to the *L* neurons.(7) Estimate the weights of the output layer by ELM approach using *Z* and vectors ${\vec{y}}_{k}$.**Update Phase (Input: single data sample**$\left({\vec{x}}_{t},y\left(t\right)\right)$:(8) Update L clouds and evolving new ones on demand (due to rule evolution conditions) in the first layer using extended evolving ADPA approach (→$L_{s,\mathrm{upd}}$ clouds).(9) Calculate (9) to achieve discretized value $y_{r}\left(t\right)$.(10) Update the feature weights $\vec{w}$ by updating the within- and between-class scatter matrices using $\left({\vec{x}}_{t},y_{r}\left(t\right)\right)$ for each feature left out and recalculating (10).(11) Perform Steps (2), (3) and (4).(12) Calculate the degree of change in all neurons.(13) Calculate the regression vector $z({\vec{x}}_{t})$.(14) Update the weights of the output layer by (5) using y(t).



The interesting computational complexity of ENFS-Uni0-reg includes the number of computations (flops) required to process one single sample through the update algorithm (second part in Algorithm 1), because this affects the on-line speed of the algorithm. The ADPA algorithm has a complexity of O(p) with *p* being the dimensionality of the input space when updating with a single sample. Constructing the neurons from the clouds takes O(mp) complexity with *m* the number of clouds extracted (the model applies in each cloud the fuzzy operator over the inputs). The definition of weights by the FWSCR technique is $Kp^{3}$ where *K* is the number of classes, because the between and within-class scatter matrices need to be updated for each class, having a complexity of $O(p^{2})$ because the matrices have a size of p×p, but this needs to be performed for each feature separately and independently. Calculating the neuron activation levels has a complexity of O(mp), as for each sample the activation levels to all *m* neurons (with dimensionality *p*) need to be calculated. The complexity of the output layer neuron is also of $O(mp^{2})$ because of the (weighted) RFWLS approach (per rule separately), requiring this complexity, as analyzed in [22]. Thus, the final complexity of ENFS-Uni0-reg for updating the model with a single sample is $O\left(p+mp+Kp^{3}+mp+mp^{2}\right)\approx O\left(mp^{2}+Kp^{3}\right)$ (as mp and *p* are ‘covered’ by $mp^{2}$).

## 5. Experiments

This paper aims to demonstrate the ability of evolving fuzzy neural networks in the interpretability of regression problems. To this end, some experiments and comparisons with state-of-the-art models will be carried out and discussed. The evaluation of the model’s performance in regression problems will be realized by the root mean square error (RMSE) expressed in (13) for the comparison of batch results and the accumulated one-step ahead prediction MAE in (15) for the results in trend lines, where *y* is the target, $\hat{y}$ is the model’s estimated value, and *N* is the number of samples.

(13) $$\mathrm{RMSE}=\sqrt{\frac{\sum_{q=1}^{N}{\left(y_{q}-{\hat{y}}_{q}\right)}^{2}}{N}}.$$

(14) $$\mathrm{MAE}=\frac{1}{N}\sum_{q=1}^{N}\left|y\left(q\right)-{\hat{y}}_{q}\right|$$

(15) $$\mathrm{MAE}\left(N\right)=\frac{\left(N-1\right)\mathrm{MAE}\left(N-1\right)\mathrm{range}\left(y\right)+|y\left(N\right)-\hat{y}\left(N\right)|}{N*0.35em\mathrm{range}(y)}$$

where MAE(0) = 0. This results in an accumulated mean absolute error (MAE) (normalized by the range of the target to achieve a kind of percentual deviation measure comparable among different data streams (with different target value ranges)). This is also in accordance with the interleaved-test-and-then-train evaluation scenario, which is widely used in the data-stream-mining and machine-learning community [45]. All values presented in the table tests in parentheses demonstrate the standard deviation of the experiments. All tests were run on a computer with the following settings: Intel(R) Core(TM) i7-6700 CPU 3.40 GHz, 16 GB RAM.

### 5.1. Intrusion Detection Using a Wireless-Sensor-Network Dataset

This study uses a dataset obtained synthetically through Monte-Carlo simulations. The method for generating the data set as well as a detailed description of the generation process is described in [46]. The data set has five columns. The first four columns are characteristics (i.e., area, sensing range, transmission range, number of sensor nodes) and the last column is the predictor or target variable (i.e., the number of barriers). The dataset generated in the aforementioned paper considers a finite number of sensors (N), uniformly and randomly distributed in a rectangular space. Each sensor is assumed to be homogeneous, i.e., the detection, transmission and computation capabilities are identical for each sensor. The dimensions of the network deployment vary from 100 × 50 m${}^{2}$ to 250 × 200 m${}^{2}$.

Some elements can be displayed about the dataset used in this experiment. Table 1 summarizes the main statistics of each feature of the dataset with numerical values, such as mean, standard deviation, maximum, and minimum value.

**Figure 4:**
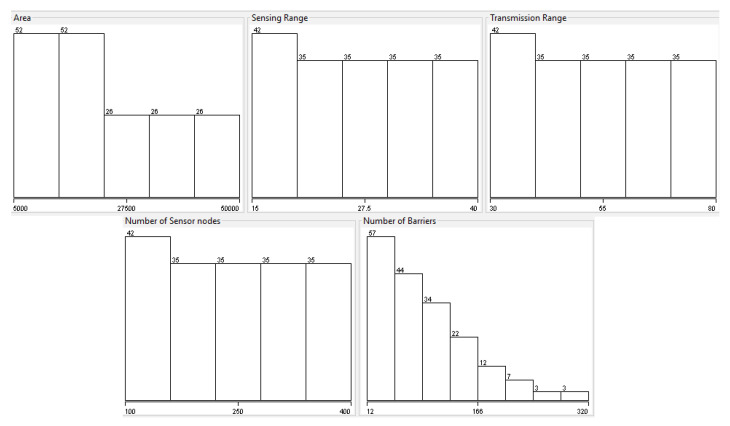
Graphical evaluation of the main features of the evaluated dataset.

A graphical evaluation of the dataset is presented in Figure 4.

Recent works using this data set investigated solutions for the same problem using automated machine learning (AutoML) [14]. However, knowledge extraction on the data set in question was not presented so far.

### 5.2. Models Used in the Experiments

The models used in the test are presented below. For appropriately eliciting the parameters (when necessary), we used a 10-fold procedure with cross validation after a 70%/ 30% split into training and separate test data, the latter serving as final evaluation set to report the expected generalization errors of the models. The following approaches were used for performance comparison:

Evolving neuro-fuzzy system based on logic neurons for regression problems (ENFS-Uni0-reg)—the evolving fuzzy neural network is used as a reference in this study. It uses an or-neuron, and the non-regularized approach was adopted to analyze all rules. A random-weighted version of the first layer of the model will also be presented in the test ((ENFS-Uni0).

MEEFIS—A multilayer ensemble evolving a fuzzy inference system primarily based on an ensemble of multiple-input multiple-output first-order evolving fuzzy inference systems (EFISs) prepared in a multilayered architecture. The parameters are the number of layers (default: 3), the number of training epochs (default: 3), and the forgetting factor (default: 0.1) [47].

ALMMo—A neuro-fuzzy approach for autonomous zero-order multiple models learning with embedded pre-processing that improves the classifier and the approximation model accuracy by creating robust models. The essential parameters are the density threshold (responsible for adding a new rule and, thus, the resulting final granularity) and ω (initialization of the size of the covariance matrix for a new rule) [48].

PSO-ALMMo—Version of ALMMo with particle swarm optimization. The parameters are maximum iteration number for the PSO algorithm (default: 200), the population number for the PSO algorithm (default: 100), inertia weight for the PSO algorithm (default: 0.7298), c1 for PSO (default: 1.49618), c2 for PSO (default: 1.49618), and the damping coefficient for PSO (set to 1) [49].

The results of regression experiments are presented in Table 2. For the regression experiment, 70% of the samples were destined for the training of the model and 30% of the samples for the test and evaluation of the model.

**Figure 5:**
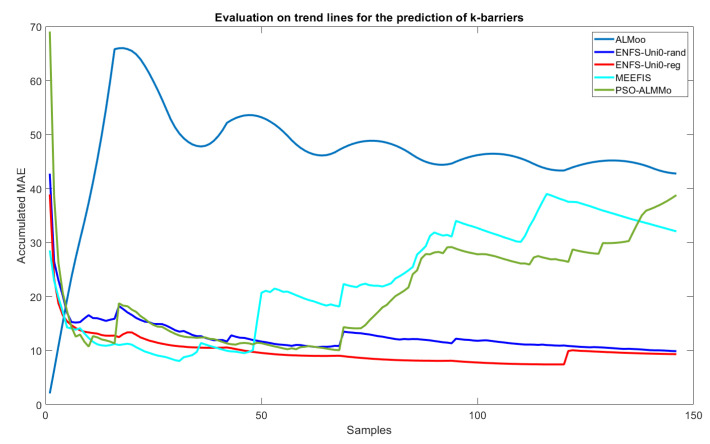
Result in trend lines for the evaluation of the k-barriers prediction.

**Figure 6:**
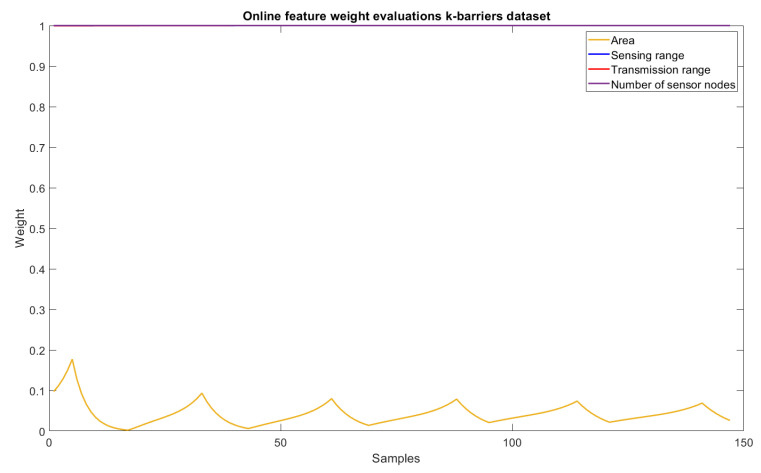
Result in trend lines for the evaluation of the k-barriers prediction.

**Figure 7:**
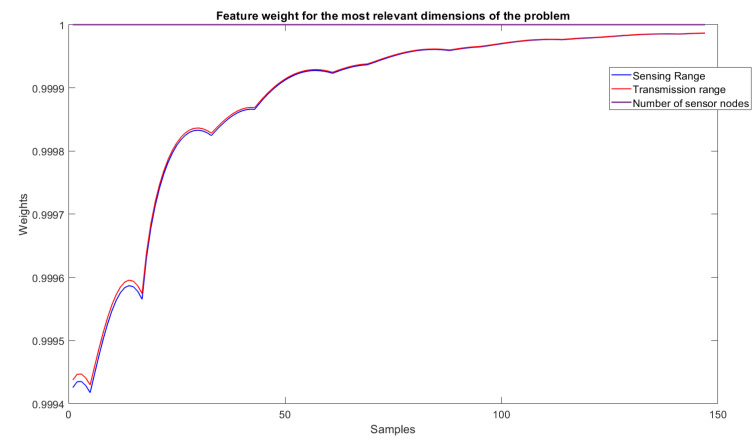
Result in trend lines for the evaluation of the k-barriers prediction (best dimensions).

**Figure 8:**
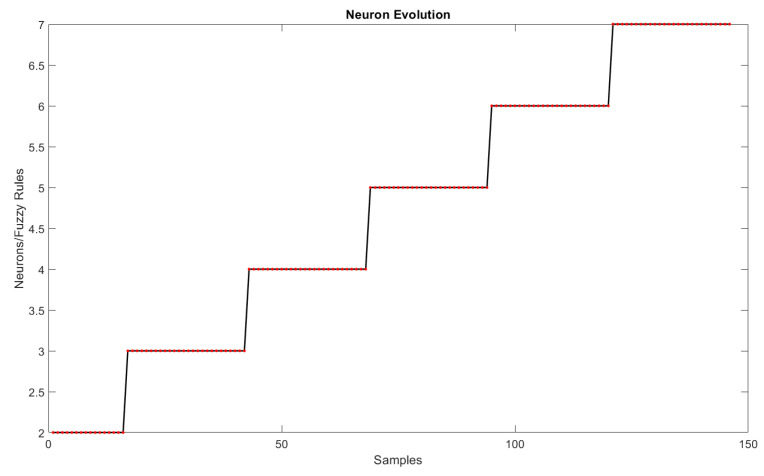
Evolution of neurons throughout the evaluation.

As for the trend-line evaluation, 20% of the samples were used for training and the architectural definition of the model, and the rest for its evaluation. The result obtained in the experiment is shown in Figure 5. The behavior of the weights related to each of the dimensions of the problem are shown in Figure 6 and Figure 7, and the evolution of neurons during the experiment can be seen in Figure 8.

## 6. Discussions

The discussions carried out in this paper seek to highlight a relationship between the main interpretability characteristics of an evolving fuzzy system model and the results obtained in the experiments performed.

### 6.1. Model Efficiency

The model presented in this paper obtained excellent results compared to state-of-the-art models of evolving fuzzy systems predicting the number of barriers. Table 2 presents the results of running 30 iterations of the algorithms, demonstrating that the proposed technique has better average RMSE performance during the experiment. There is also a gain in efficiency using the feature weights technique compared to generating random weights. The best-performing results of the ENFS-Uni0-reg model were observed in the data-stream test presented in Figure 5. The model maintained the curve with the lowest MAE values throughout large parts of the stream (only at the beginning did all approaches seem to behave similarly), and this with a largely monotonically decreasing trend, which was not the case for the related SoA works (which showed significantly increasing trends from some samples onwards).

### 6.2. Distinguishability and Simplicity

The criterion of simplicity was met in this paper because the model applied simple, coherent, and efficient techniques to solve the problems. The results achieved by the proposed model were the most assertive when compared to the state-of-the-art approaches to the prediction of evolving fuzzy systems for regression problems, and this with a small number of rules (maximal seven at the end of the learning process). Table 2 and Figure 5, in combination with Figure 8, support these conclusions.

**Figure 9:**
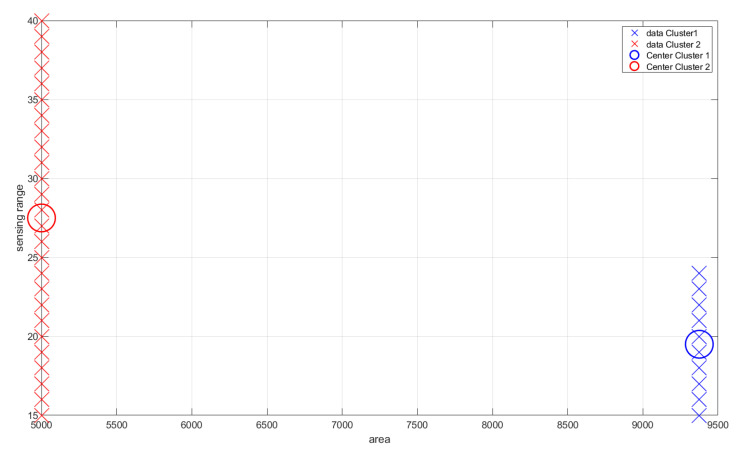
Initial result of data fuzzification (relation area versus sensing range).

**Figure 10:**
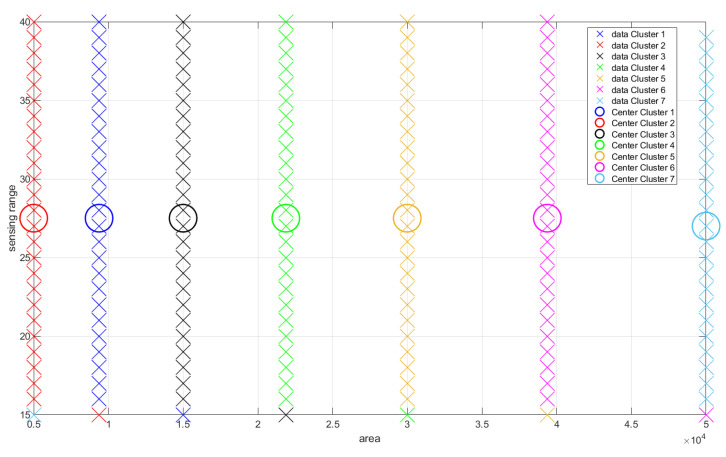
Final result of data fuzzification (relation area versus sensing range).

On the other hand, the distinguishability criteria are met by the Gaussian neurons of the first layer of the model, which are separable and entirely distinguishable. Figure 9 presents the first configuration of the fuzzy rules found through clustering (the one that defines the initial architecture of the model in the offline phase of the ADPA model), and Figure 10 presents the final version of the model’s rules after the stream learning process. In both cases, the distinct clusters contained in the data (marked by crosses in different colors) are one-to-one represented by rules (thus meeting distinguishability).

### 6.3. Consistency

**Figure 11:**
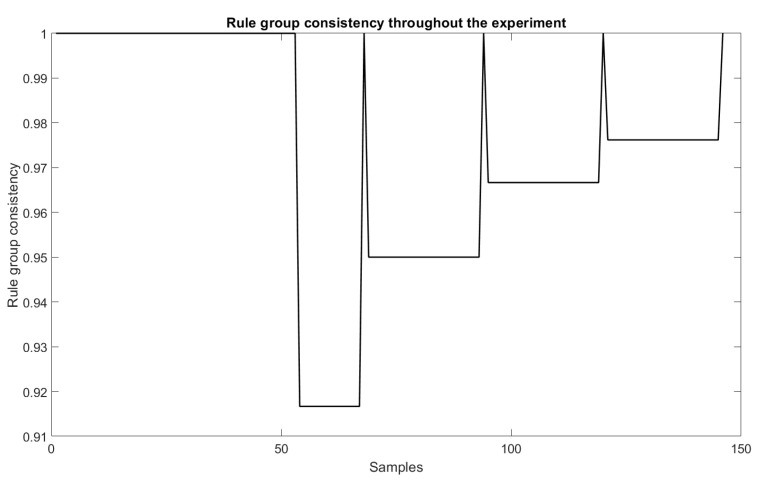
Consistency of fuzzy rules during evaluation.

The consistency of fuzzy rules can be identified through the relationship between their antecedents and rule consequents. In this paper, to examine this condition, the following formulation proposed in [12] was used: A rule $z_{n}$ is inconsistent with $z_{n+1}$ if and only if the similarity of the consequents of these rules is greater than or equal to the similarity of the antecedents of them, with rule antecedents being greater than a single threshold, defined here as 0.8 (as in [12]). This evaluation condition can be defined by [12]:

(16) $$\begin{array}{c} Rule\;z_{1}\;is\;inconsistent\;to\;Rule\;z_{2}\;if\;and\;only\;if\\ S_{\mathrm{ante}}\left(z_{1},z_{2}\right)⩾S_{\mathrm{cons}}\left(z_{1},z_{2}\right)\;with\;S_{\mathrm{ante}}\left(z_{1},z_{2}\right)⩾0.8. \end{array}$$

where $S_{\mathrm{ante}}$ and $S_{\mathrm{cons}}$ close to 1 invariably can be assumed to indicate a heightened similarity, and when they are close to 0, a low similarity [12]. Similarities of rule antecedents and consequents were performed according to the criteria established in [12]. Therefore, a target rule is considered consistent (receiving the value 1) when it meets the criteria established in Equation (16). Otherwise, this rule is inconsistent and receives the value 0 to represent this factor. Figure 11 presents the evaluation of this criterion for the target experiment based on the values obtained in the evaluation of the similarity of the antecedents and the consequents. The criterion evaluation to generate the graph involves the average of the consistencies of the group of fuzzy rules generated at each model evaluation moment.

This experiment compared the consistency with the group of fuzzy rules generated in each of the evaluation moments of the samples. It can be noted that, until the evaluation of sample 54, the rules were consistent. After that, one of the rules remained inconsistent with the group of fuzzy rules generated until the evaluation of sample 67. This behavior occured other times. However, the model manages to perform the consistency adjustment so that, in the end, the group of seven rules consistently follows the criterion established in Equation (16).

### 6.4. Feature Importance Levels

The features relevance criteria for the problem were solved with the new approach proposed in this paper. Now, it is possible to differentiate the relevance of the data set features to identify the separation between responses, with many barriers necessary for identifying intruders from those that do not need so many barriers for the same purpose. Figure 6 highlights the results throughout the evaluations and allows the conclusion that the area dimension has little relevance to separation between the expected responses. The other dimensions, however, remain extremely relevant (weight values close to 1). In this case, it is possible to conclude that the number of sensor nodes, sensing range, and transmission range are the dimensions that best define whether a high or a low number of barriers should be applied to detect intruders.

### 6.5. Interpretation of Consequents

The values of their consequents measure the relevance of fuzzy rules in their output contributions. As they are crisp values, they can express the relevance of a rule to find an expected output or how that rule strongly contributes to the prediction of the value. In evaluating the values present in Table 3, we can identify that rule 1 is the one that most positively contributes to the increase in the values to be predicted. Likewise, when rule 2 is triggered, it contributes to decreasing the values predicted by the neural network in the third layer. In this sense, when a sample falls into a rule, i.e., for the rule activation level it is higher (close to 1), its consequent value can be used to find the reason for the final prediction (based on the corresponding rule antecedent), weighted together with the other reasons due to the consequents of the other rules and their activation levels (this is because a weighted average over rule consequents is produced for final predictions). Hence, the consequents in combination with the corresponding rule antecedents can serve as output (prediction) explanations for new samples.

In addition to the relevance aspects that the model has when creating a weight for the fuzzy rules, the ENFS-Uni0-reg also has a sample-by-sample evaluation criterion showing whether the fuzzy rules have changed over stream samples and to what extent. In this evaluation, it is possible to identify the impact of new samples on the fuzzy rules in the evolving training. Table 4 presents an example of this evaluation.

In this model update round, of the seven fuzzy rules that are part of its architecture, only five were changed, four of them with a decrease in the consequent value and one with an increase (compared to the previous version of the fuzzy rule before the update).

**Figure 12:**
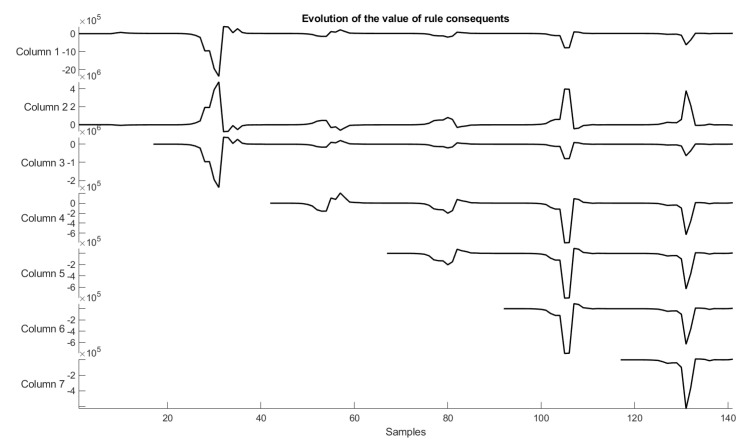
Evolution of rule consequents.

Another factor that can help in the evaluation of rule consequences is connected to their evolution. Figure 12 represents the evolution of the consequent values in the rules generated by the model.

### 6.6. Knowledge Expansion

Knowledge expansion occurs through the fuzzy rules obtained throughout the experiment. The evolution of fuzzy rules presented in Figure 8 demonstrates that the model, upon receiving a new sample, identified a new behavior and defined a new meaningful relationship for predicting the number of barriers. Presenting the logical relations obtained in the data set can also be seen as an expansion of knowledge, since this work is the first to present the linguistic relations for solving this problem in the format of IF-THEN rules. Another way of evaluating the knowledge obtained is through evolution and changes in fuzzy rules. For the first batch of samples, only two fuzzy rules were needed to determine the number of sensor barriers. These rules are presented below.

**Rule 1.**—If (area is high) with impact 0.11 or (sensing range is high) with impact 0.99 or (transmission range is high) with impact 0.99 or (number of sensor nodes is high) with impact 1.00 then (number of barriers is −1971.89).

**Rule 2.**– If (area is small) with impact 0.11 or (sensing range is small) with impact 0.99 or (transmission range is small) with impact 0.99 or (number of sensor nodes is small) with impact 1.00 then (number of barriers is 2061.66).

The evolution of the rules can be seen as a knowledge gain and expansion. The seven fuzzy rules obtained at the end of the training are also presented below.

**Rule 1.**—If (area is small) with impact 0.02 or (sensing range is medium) with impact 0.99 or (transmission range is medium) with impact 0.99 or (number of sensor nodes is medium) with impact 1.00 then (number of barriers is 15,937.14).

**Rule 2.**—If (area is small) with impact 0.02 or (sensing range is small) with impact 0.99 or (transmission range is small) with impact 0.99 or (number of sensor nodes is small) with impact 1.00 then (number of barriers is −51,137.92).

**Rule 3.**—If (area is small) with impact 0.02 or (sensing range is medium) with impact 0.99 or (transmission range is high) with impact 0.99 or (number of sensor nodes is high) with impact 1.00 then (number of barriers is 5260.04).

**Rule 4.**—If (area is small) with impact 0.02 or (sensing range is medium) with impact 0.99 or (transmission range is high) with impact 0.99 or (number of sensor nodes is very high) with impact 1.00 then (number of barriers is 8603.60).

**Rule 5.**—If (area is medium) with impact 0.02 or (sensing range is medium) with impact 0.99 or (transmission range is high) with impact 0.99 or (number of sensor nodes is extremely high) with impact 1.00 then (number of barriers is 6352.77).

**Rule 6.**—If (area is high) with impact 0.02 or (sensing range is medium) with impact 0.99 or (transmission range is high) with impact 0.99 or (number of sensor nodes is exceeding high) with impact 1.00 then (number of barriers is 7258.79).

**Rule 7.**—If (area is very high) with impact 0.02 or (sensing range is medium) with impact 0.99 or (transmission range is high) with impact 0.99 or (number of sensor nodes is excessively high) with impact 1.00 then (number of barriers is 7593.08).

Another way of interpreting knowledge that also meets the criteria for evaluating the dimensions of the problem is the ability to create reduced rules based on the evaluation of the weights of rule antecedents. As the area dimension has a much lower value weight than the others, it can be suppressed from interpreting the results as it does not contribute at all to identifying a small or large number of barriers. An example of a reduced fuzzy rule (Rule 1 at the end of the experiment) can be seen below.

**Rule 1.**—If (sensing range is medium) with impact 0.99 or (transmission range is medium) with impact 0.99 or (number of sensor nodes is medium) with impact 1.00 then (number of barriers is 15,937.14).

Another way of confirming the expansion of knowledge is linked to the validation of the results extracted by specialists on the subject (Appendix A). In this case, the seven fuzzy rules generated by the model were submitted for validation by the main owner responsible for the elaboration and dissemination of the target data set (see [46]) of this study. In response, positive feedback was obtained on the plausibility of the rules for the context of identifying the number of barriers. The following rules were presented to the owner of the data set (and expert in this application scenario), including the transformation of rule consequents into linguistic terms:

**Rule 1.**—If (area is small) with impact 0.02 or (sensing range is medium) with impact 0.99 or (transmission range is medium) with impact 0.99 or (number of sensor nodes is medium) with impact 1.00 then (number of barriers needed to increase strongly).

**Rule 2.**—If (area is small) with impact 0.02 or (sensing range is small) with impact 0.99 or (transmission range is small) with impact 0.99 or (number of sensor nodes is small) with impact 1.00 then (number of barriers needed to decrease).

**Rule 3.**—If (area is small) with impact 0.02 or (sensing range is medium) with impact 0.99 or (transmission range is high) with impact 0.99 or (number of sensor nodes is high) with impact 1.00 then (number of barriers needed to increase).

**Rule 4.**—If (area is small) with impact 0.02 or (sensing range is medium) with impact 0.99 or (transmission range is high) with impact 0.99 or (number of sensor nodes is very high) with impact 1.00 then (number of barriers needed to increase).

**Rule 5.**—If (area is medium) with impact 0.02 or (sensing range is medium) with impact 0.99 or (transmission range is high) with impact 0.99 or (number of sensor nodes is extremely high) with impact 1.00 then (number of barriers needed to increase).

**Rule 6.**—If (area is high) with impact 0.02 or (sensing range is medium) with impact 0.99 or (transmission range is high) with impact 0.99 or (number of sensor nodes is exceedingly high) with impact 1.00 then (number of barriers needed to increase).

**Rule 7.**—If (area is very high) with impact 0.02 or (sensing range is medium) with impact 0.99 or (transmission range is high) with impact 0.99 or (number of sensor nodes is excessive high) with impact 1.00 then (number of barriers needed to increase).

The owner could confirm these rules as valuable and interesting, as can be seen in the email exchange letter shown in Appendix A.

## 7. Conclusions

This paper presented the solution to the problem of identifying k barriers used for intrusion detection using data from wireless sensor networks with interpretability aspects. In this context, the model proposed in this paper acted assertively and interpretably in extracting knowledge about the analyzed problem.

The results obtained and compared with state-of-the-art models in evolving fuzzy systems support the idea that the ENFS-Uni0-reg model can help in defining the number of barriers needed to identify intruders in a given territory. The model proposed in the paper proved to be quite efficient in the approach, as it improved the RMSE by around 10% in the prediction of barriers compared to the model ENFS-Uni0 (Table 2). This improvement is accentuated when the results are compared to the other models evaluated in the experiment (21.86%, 142.38%, and 117.47%, compared with MEEFIS, ALMMo, and PSO-ALMMo, respectively). In Figure 5, there is also a sizeable numerical difference between the results obtained by the model throughout the evaluations. This allows us to conclude that the model remained stable throughout its evaluation, predicting values closer to those expected compared to the state-of-the-art model evaluated in the experiment.

The results obtained through model interpretability demonstrate how the data behave over time and allow for a sample-by-sample evaluation of the problem that seeks to identify the required number of barriers for territorial protection using a wireless sensor network. Thus, the costs of prediction and installation of equipment in these regions can be minimized.

The fuzzy rules extracted can be seen as a novel way to understand the behavior of the underlying problem, facilitating the dissemination of knowledge about the problem studied. These fuzzy rules can facilitate the construction of expert systems on the topic. The if-then rules can be easily adapted in computer systems with programming languages, and this explicit knowledge can help professionals who want to act in the prediction of the number of barriers and do not have much knowledge about the topic.

The limitations found in this approach are connected to a reference value for the process of discretization of regression values. This paper used the average value of the number of barriers. Perhaps a human expert can better contribute to the definition of a more fair value to discretize the number of barriers to this problem.

Future work may address improvements in the ENFS-Uni0-reg architecture, as well as new training models and optimization techniques. Another possible extension to this work is to implement an intelligent system based on rules based on the knowledge acquired in this research. 

## Figures and Tables

**Table 1:** Statistics on the dataset for the number of k-barriers prediction.

Feature	Mean	Standard Deviation	Maximum	Minimum
area	24,375.00	15,197.25	50,000.00	5000.00
sensing range	27.50	7.52	40.00	15.00
transmission range	55.00	15.04	80.00	30.00
number of sensor nodes	250.00	90.24	400.00	100.00
number of barriers	94.07	65.17	320.00	12.00

**Table 2:** Result of wireless sensor network data set (highlighted in bold is the best result).

Model	RMSE.
ENFS-Uni0-reg	**11.16 (2.21)**
ENFS-Uni0	12.29 (4.81)
MEEFIS	13.60 (3.91)
ALMMo	27.05 (1.67)
PSO-ALMMo	24.27 (5.07)

**Table 3:** Values of the consequents of the generated fuzzy rules.

Fuzzy Rule	v	v Standardized
1	15,937.14	0.6996
2	−51,137.92	−2.2412
3	5260.04	0.2314
4	8603.60	0.3780
5	6352.77	0.2794
6	7258.79	0.3191
7	7593.08	0.3337

**Table 4:** Interpretability with respect to (degree of) changes in fuzzy neurons during the evolving phase.

Rule 1 did not change.
Rule 2 did not change.
Rule 3 changed with one membership function, changing the rule consequent from positive to negative.
Rule 4 changed with one membership function, changing the rule consequent from positive to negative.
Rule 5 changed with one membership function, changing the rule consequent from positive to negative.
Rule 6 changed with one membership function, changing the rule consequent from negative to positive.
Rule 7 changed with one membership function, changing the rule consequent from positive to negative.

**Figure A1:**
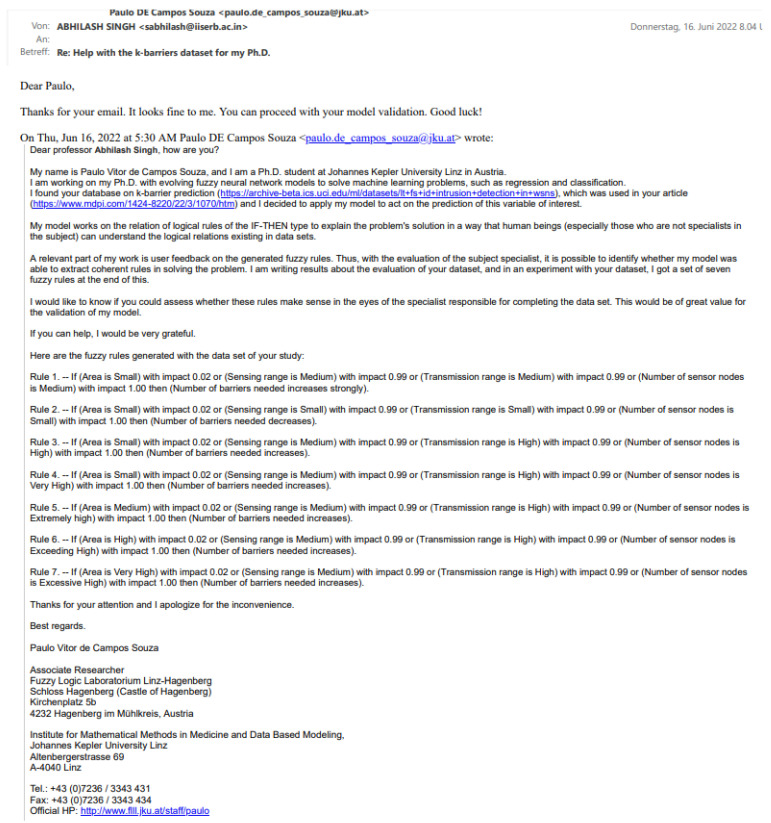
Confirmation email about the consistency of the generated fuzzy rules.

## Data Availability

Publicly available datasets were analyzed in this study. This data can be found here: https://archive-beta.ics.uci.edu/ml/datasets/lt+fs+id+intrusion+detection+in+wsns.

## Abbreviations

The following abbreviations are used in this manuscript:

ENFS-Uni0-reg	Evolving neuro-fuzzy system based on logic neurons for regression problems.
ENFS-Uni0	Evolving neuro-fuzzy system based on logic neurons.
EFNNs	Evolving fuzzy neural networks
EFS	Evolving fuzzy systems
FWSCR	Feature weight separability criteria for regression problems.
RMSE	Root mean square error.
MAE	Mean absolute error.
MEEFIS	Multilayer ensemble evolving fuzzy inference system.
EFISs	Evolving fuzzy inference systems.
ALMMo	Autonomous zero-order multiple learning.
PSO-ALMMo	Version of autonomous zero-order multiple learning with particle
	swarm optimization.
PSO	Particle swarm optimization.
RWLS	Recursive weight least squares.
AutoML	Automated machine learning.
LOFO	Leave-one-feature-out.

## Appendix A

The confirmation of the usefulness of the generated fuzzy rules (Figure A1 was performed by the owner of the data set used in this study. The following link provides more information about the owner: https://www.abhilashsingh.net/. Accessed on 16 June 2022.
